# Setting life cycle assessment (LCA) in a future-oriented context: the combination of qualitative scenarios and LCA in the agri-food sector

**DOI:** 10.1186/s40309-022-00203-9

**Published:** 2022-06-27

**Authors:** Ariane Voglhuber-Slavinsky, Alberto Zicari, Sergiy Smetana, Björn Moller, Ewa Dönitz, Liesbet Vranken, Milena Zdravkovic, Kemal Aganovic, Enno Bahrs

**Affiliations:** 1 Competence Center Foresight, Fraunhofer Institute for Systems and Innovation Research ISI, Breslauer Straße 48, 76139 Karlsruhe, Germany; 2 Institute of Farm Management, University of Hohenheim, Schwerzstraße 44, 70599 Stuttgart, Baden-Württemberg Germany; 3 Division of Bioeconomics, Department of Earth and Environmental Sciences, KU Leuven, Celestijnenlaan 200E box 2411, 3001 Leuven, Belgium; 4 DIL German Institute of Food Technologies e.V, Prof.-von-Klitzing-Str. 7, D-49610 Quakenbrück, Germany

**Keywords:** Foresight, Qualitative scenarios, Life cycle assessment, Background system, Agri-food system

## Abstract

By combining qualitative scenarios and life cycle assessment (LCA), we place the latter in a larger context. This study outlines the importance of the integration of future perspectives into LCA, and also the significance of taking changes in the environment of technology into account, rather than just technological development itself. Accordingly, we focused on adapting the background system of an attributional LCA in the agri-food sector. The proposed technology was assumed not have evolved in the considered time horizon. In this context, the objectives of this paper were twofold: (i) to methodologically prove the applicability of integrating qualitative scenarios into LCA and (ii) to focus on changes in the background system, which is sometimes overlooked in the context of future-oriented LCA. This allowed to evaluate the future potential of different technologies, assessing their environmental impact under uncertain future developments. Methodologically, the qualitative information from scenarios was transformed into quantitative data, which was successively fed into the life cycle inventory (LCI) of the LCA approach. This point of integration into the second phase of LCA translates into future changes in the entire environment in which a technology is used. This means that qualitatively described scenario narratives need to be converted into value estimates in order to be incorporated into the LCA model. A key conclusion is that changes in the background of an LCA—the changing framework expressed through the inventory database—can be very important for the environmental impact of emerging technologies. This approach was applied to a food processing technology to produce apple juice. The proposed methodology enables technology developers to make their products future-proof and robust against socioeconomic development. In addition, the market perspective, if spelled out in the scenarios, can be integrated, leading to a more holistic picture of LCA with its environmental focus, while simultaneously empowering actors to make the right strategic decisions today, especially when considering the long investment cycles in the agri-food sector.

## Introduction

The agri-food system will face many challenges in the years to come due to factors such as climate change, a growing world population, changes in consumer demand, and the dissemination of digitalization [1]. The United Nations’ Sustainable Development Goals (SDGs), the Paris Climate Agreement, the Farm to Fork Strategy, and the Biodiversity Strategy for 2030 point out the need to transform the management of food systems into a more sustainable process concerning the environmental, economic, and social dimensions [2–5]. In addition, the COVID-19 pandemic situation has highlighted the importance of resilient food systems. Anderies et al. [6] saw sustainability as the main goal, while resilience can contribute to reaching this goal. “Resilience then gives us information about what might need to happen at the system-level for sustainability to improve” [7]. In many ways, these two concepts are interlinked. Agrobiodiversity can be an indicator of resilient food systems [8], and at the same time, sustainable agricultural cultivation needs to guarantee the management of biodiversity. However, how can these concepts be followed or implemented?

Life cycle sustainability assessment (LCSA) can be applied in order to measure the sustainability performance of food products or production and to identify the areas with the greatest impact on sustainability [9–14]. LCSA is an approach of the UNEP/SETAC Life Cycle Initiative to combine life cycle assessment (LCA), life cycle costing (LCC), and social life cycle assessment (S-LCA) and is based on the ISO 14040 guidelines [15]. While LCSA integrates social [16], environmental, and economic aspects, life cycle assessment (LCA) solely focuses on the environmental dimension of sustainability [17], providing “insights on the environmental impacts of products and services over their lifecycle” [18, 19]. The methodology is specified in the ISO 14040/14044 framework, while the principles and framework conditions are addressed in ISO 14040 and the requirements in ISO 14044 [20, 21]. According to the definition given by the International Organization for Standardization, “LCA is the compilation and evaluation of the inputs and outputs and the potential impacts of a product system throughout its life cycle” [20]. It is therefore a methodology used to assess the potential environmental impacts of a particular product, process, and/or service throughout its entire life cycle [22]. Ecological aspects such as climate change, freshwater use, occupation and changing of land use, aquatic eutrophication, toxic impacts, and the use of nonrenewable resources [23] are included in the assessment. LCC, on the contrary, can be used to look at the cost implications of acquisition, transport, installation, operation and maintenance, disposal, and residual value [24]. Human rights, working conditions, health and safety, governance, etc. are social and socioeconomic impacts of a product’s life cycle that can be assessed via S-LCA [25].

Although LCA techniques are often used for strategic purposes and long-term planning, they traditionally deal with present or near-future conditions, e.g., alternative options of a product comparing the studied unit to a reference unit. Foresight methods can help to integrate the time perspective into LCA techniques by considering changes of the model parameters in the future. In particular, considering the fast pace development in the agri-food sector [1, 26–30], as well as the need to meet the SDGs and to create a sustainable and resilient food system, future developments have to be integrated into the assessment of different options within the food system [31]. This includes qualitative uncertainties such as how widespread the technology is that is going to be used [32]. Thonemann et al. [33] conducted a literature review on prospective LCAs and defined uncertainty as the main challenge to overcome. Therefore, further research is needed to develop appropriate approaches to include uncertainty analyses in prospective LCA. The uncertainty evolving from the complex and interactive nature of the framework conditions or future scenarios cannot be reduced. The focus here should rather be on acknowledging this kind of uncertainty and using the different futures to “outline and better inform directions for action” [34]. Furthermore, the identification of robust options, withstanding different future developments, can be a benefit of the integration of future perspectives. For the formation of strategic orientation, different foresight methods and particularly scenario development can be applied in sustainability assessment. Active engagement with the future helps to take various influencing factors within the food system into consideration and assists in finding feasible options in different future worlds [35, 36].

LCA allows to consider all stages of the agri-food supply chain and can therefore provide a comprehensive view. There is a considerable amount of literature reflecting on the impact of damage occurring to ecosystems and biodiversity on agricultural production, highlighting the importance of the environmental dimension [37–40]. Nature and ecosystems are the basis for food production, and, at the same time, they can be deteriorated or promoted depending on the kind of management exercised. The framework of ecosystem services (ESS) mentioned in this context was developed in order to make visible the various advantages that ecosystems provide to humans [41].

It is becoming increasingly important to integrate future needs, threats, opportunities, and challenges into the assessment of products or production methods. On the one hand, the complexity of international value chains and, on the other hand, the manifestation of weak signals into impelling trends or even the occurrence of significant events call for a systemic and future-oriented approach. First, this serves to identify different points of change and to take a systematic perspective. Second, LCA provides a structured approach for identifying hotspots along the supply chains of products. Based on this, the view can be broadened toward future options. In this aspect, the combination of qualitative scenarios and quantitative methods can help to address challenges in the agri-food system in a systematic manner. This results in a future-proof analysis of the life cycle of products enabling engineers to develop more resilient and sustainable technologies meeting the demands of the market. The consideration of qualitative aspects and alternative future developments in LCA puts this quantitative method into a broader context.

This study describes how qualitative aspects of future scenarios can be combined with the quantitative values used in LCA to calculate the environmental impact of products or processes. In “State of the art—future-oriented life cycle assessment,” we provide an overview of the current discussion of prospective or future-oriented LCA approaches and describe the specific characteristics of LCA in the agri-food sector. The actual approach of the quantification of qualitative descriptions is presented in “Methodology,” as well as the difference between conventional apple juice and apple juice treated with the technology developed in the FOX project^1^ (hereafter called “FOX apple juice”) and its effects on the life cycle inventory (LCI).^2^ “An integral part of an LCA is the production of a life cycle inventory, listing the inputs and outputs associated with the product system under study” [43]. “Results” describes the results, and the discussion and conclusions are presented in “Discussion” and “Conclusion,” respectively.

In particular, we wanted to answer the following research questions:

1. How can future framework conditions influence the results of LCA in general, and, in particular, how does the environmental impact of FOX apple juice change in different futures?
2. How can qualitative scenarios be systematically combined with LCI parameters to account for changes in future framework conditions, and what are the methodological challenges within this approach?
3. Where can the most important environmental impacts be detected along the supply chain?

The objectives of this study were to test the combination of qualitative and quantitative research. The research project FOX served as a test case for this combination. Within the framework of the project, small processing plants for fruit and vegetables are being developed, which, among other things, produce apple juice. LCA for the technologies, as well as the future framework scenarios for the European food sector, elaborated in the project was used as the basis for the combination of methods.

The proposed framework should be useful for LCA practitioners to integrate different futures in the assessment of technologies under development. Hotspots of environmental impact can be identified when assessing the differences of incumbent technology and the innovations in the different futures. Furthermore, there is the possibility to alter product characteristics and derive the difference that this makes in alternative futures.

## State of the art—future-oriented life cycle assessment

In this section, we provide an overview of the current discussion of prospective or future-oriented LCA approaches and describe the specific characteristics of LCA in the agri-food sector. As already mentioned, most LCA studies base their results on current values and can therefore only make assumptions for the present situation [18, 19, 32]. In particular, the assessment of products or technologies in the development stage should take future perspectives into consideration; as in early stages, decisions on the outlay of a product or process are important cornerstones for future success [44, 45]. Since 2014, there has been a rapid rise in articles on the topic of prospective LCAs of emerging technologies [33].

Based on a review of the literature combining these two approaches, there are several terms used to describe the integration of future aspects into LCA, such as prospective, consequential, dynamic, anticipatory, and ex ante [19, 33, 34, 46, 47]. Olsen et al. [32] added the term change-oriented. In the first publications within the field of future-oriented LCA, the term consequential LCA was also used in this sense [48]. However, according to Arvidsson et al. [46] both types of LCA, attributional and consequential, can be prospective and retrospective.

In contrast to the definition by Cucurachi et al. [19], which focuses on the upscaling of emerging technologies in the future, we wanted to look at the performance of a defined technology under different future framework conditions. Therefore, the scale of the technology stayed the same, but the framework conditions in which the technology is used changed. This means that only the background system was adjusted, as also described in the work of Mendoza Beltran et al. [34]—or as Arvidsson et al. described it [46], “prospective inventory modeling” was conducted, and the foreground system stayed the same. The background system refers to the framework conditions that a technology is embedded in, while the foreground system models the technology itself. In other words, “scenarios in prospective LCA draw from multiple databases exogenous to LCA to address future sociotechnical changes or so-called exogenous system changes” [34]. The different options of including future perspectives into LCA were also explained by van der Giesen et al. [18], who distinguished between manipulation of the foreground in contrast to adjustment of the background system, with the explicit task to “transform existing LCI databases toward future contexts” for the latter.

**Fig. 1:**
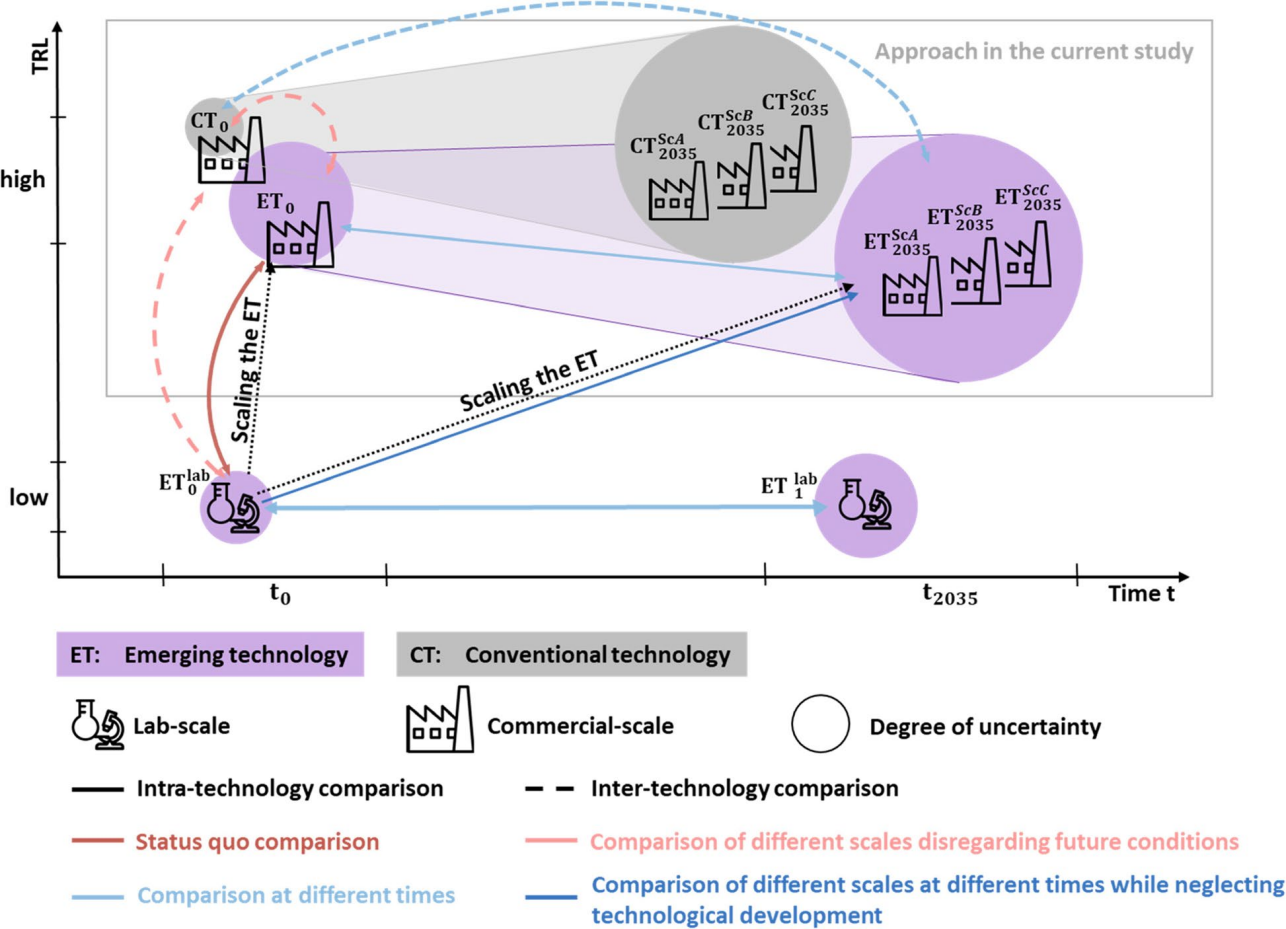
Location of the approach used within this study based on the general framework of Thonemann et al. [33] (source: Own presentation based on the work of Thonemann et al. [33])

Thonemann et al. [33] proposed a differentiation of temporal and technological aspects within the field of prospective LCA. They identified 65 studies in their literature analysis concerning the prospective LCAs of emerging technologies. On the X- and Y-axes, the time and technology readiness level (TRL) were plotted, respectively. We located our approach within this framework (see Fig. 1). Both the emerging technology (ET) and the conventional technology (CT) were set in the future context (movement from the upper left to the upper right part), leading to CT_2035_ and ET_2035_ for scenarios A–C, respectively. Other options, not followed within this study, would be to either put an ET at lab scale into the future context (lower part of the figure with movement from left to right) or to combine a change in the time scale and TRL (diagonal movement from the lower left to the upper right part of the coordinate system).

In order to integrate future perspectives into LCA, qualitative scenarios were used. The SETAC^3^ working group on scenarios, for example, lists six groups of methods to build scenarios, namely, “extrapolation methods, exploratory methods, dynamic modeling, cornerstone scenarios, participatory methods and normative methods” [32]. Within the field of future studies, however, a huge variety of different scenarios and techniques for their development are being discussed. Scenarios in their broadest sense can be described as plausible and consistent images of the future [49]. In this publication, LCA was combined with the so-called cornerstone scenarios, in line with the SETAC WG definition, knowing also that other methods can be applied to refer to the uncertain future [32]. These cornerstone scenarios cover large-scale systems [50]. Furthermore, they “better inform long-term and strategic decision making, which are fundamental characteristics of prospective LCA” [34]. Olsen et al. [32] claimed that for long-term perspectives, “scenarios established by qualified experts about future technological and economic developments are indispensable in future technology assessment.” This approach therefore addresses “the system's strategic environment,” rather than the production system itself [32]. In addition, the qualitative scenarios were elaborated in a participatory process [51], involving experts from the whole agri-food value chain [36].

Thonemann et al. [33] further defined three main challenges in the area of prospective LCA: comparability, data, and uncertainty challenges, the latter of which was further divided into parameter, scenario, and model uncertainty. We determined parameter and scenario uncertainty to be the most prominent when conducting future-oriented LCA using qualitative scenarios. Parameter uncertainty can occur when future values for certain parameters are assessed, while scenario uncertainty describes the fact that the actual future is unknown, and that only alternative developments can be discussed. Assumptions forming the basis of the scenarios are possible, but no statement of the probability can be made [51]. Buyle et al. noted that one should not interpret the results of ex ante LCA as absolute but more as an “indication of what might happen” [47]. They further explained that the question is not about being right or wrong but rather if the work enables decision-makers to actually make decisions [47].

### Specific characteristics of life cycle assessment in the agri-food sector

To estimate the sustainability of agricultural food products, LCA is usually used with its environmental focus, which can be divided into the six production stages of input, agricultural production, processing, distribution (including the transportation to the warehouse and to the retailer), consumption, and waste management (including food waste generated throughout the entire life cycle) [52]. Dijkman et al. [52] identified six stages in the LCA of agricultural products, which is in line with other characterizations of the supply chain in the agri-food system. However, in agriculture and food processing, LCA studies often concentrate on the first stages of the supply chain and are called cradle-to-farm gate studies [52]. In contrast, this study assessed the whole supply chain, including the disposal and retail stages, while excluding [52, 53] the input stage (e.g., agrochemicals) [54–57].

The selection of impact categories was very broad in this study. According to Dijkman et al. [52], who analyzed different food product LCAs “based on representativeness of their outcomes among LCA studies for similar products, and inclusion of processes beyond the farm gate,” many studies include a few impact categories only. Referring to their analysis, the often-applied impact categories are fossil or primary energy use, global warming, acidification, eutrophication, land use, photochemical ozone formation, abiotic depletion, aquatic ecotoxicity, terrestrial ecotoxicity, and human toxicity—while it has to be mentioned that in most of the analyzed studies, only a selection of the impact categories were used rather than all of them.

When considering the other advantages that agriculture provides to society besides the production of food, the choice of functional unit can be challenging, and a question arises as to how to account for land management, to provide income opportunities in rural areas, or to maintain the nutritional quality of food [52, 58, 59]. Additional methodological issues in the assessment of agricultural products are setting system boundaries between the technosphere and ecosphere, the variability of agricultural production systems, the modeling of nutrient and pesticide flows, especially concerning their interaction with local circumstances [60], and the “limited number of impact categories” [59], with the need to broaden the boundaries to include toxicity, land use, and water use-related impacts [52].

### Specific characteristics of future-oriented life cycle assessment in the agri-food sector

Some authors have addressed the topic of future-oriented LCA in the agricultural sector [61–63], with some rather focused on possible alternative developments, such as dietary changes, altered production efficiencies, reduced on-farm food losses, secondary use of byproducts, or different crop management options, such as earlier sowing, cultivar selection, and reduced use of herbicides and insecticides [64–69]. For example, Niero et al. assessed the effects of climate change on Danish spring barley cultivation using LCA [61]. In contrast to this study, Niero et al. concentrated on some key parameters in their alternative scenarios. Odegard and van der Voet [63] constructed four scenarios along four driving forces, which they quantified. The results showed resource use in agriculture in 2050 applying a kind of footprint methodology. Meanwhile, we worked with the whole system of factors, not with single factors, of future scenarios to build a narrative.

## Methodology

**Fig. 2:**
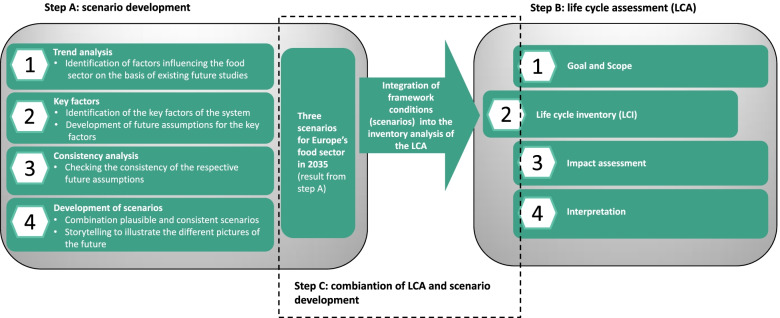
Overview of the combination of qualitative scenarios and life cycle assessment (LCA) and their respective prior development steps (source: Own presentation)

Prior to the actual approach followed within this research, two separate steps were conducted. The development of qualitative scenarios [36] and LCA was part of the H2020 Europe project FOX (FOod processing in a boX), aimed at shortening the food supply chains of a large variety of fruit and vegetables through the adoption of four innovative, flexible, mobile, and small-scaled mild processing units [70]. The individual methodological steps of the two approaches are summarized in Fig. 2.

### The life cycle assessment

The LCA, which was carried out from a consumer point of view taking into consideration all of the phases of the supply chain (production, processing, retail, consumer, and disposal), with a particular focus on the processing stage, followed a cradle-to-grave approach and ISO 14044 [20]. The focus on the processing stage was based on the fact that in the project FOX, various small-scale processing units are developed. The system boundaries were set to include all of the life cycle stages from cradle to grave, because “leaving out certain life cycle stages in an LCA could lead to an incomparability of results” [33]. In addition, the future-oriented LCA was based on the traditional LCA conducted within the project FOX [70]. The reference unit chosen was 1 L of apple juice, and the analysis was conducted with the help of Microsoft Excel® and SimaPro® (version 9.1.0.8) software, the latter of which is an environmental modeling software developed by PRé Sustainability B.V [71]. .SimaPro® allows for detailed analysis of material flows from background databases and is the most widely used software in the field of LCA. Microsoft Excel was used for data collection, because project-wide data can easily be collected without the hurdle of installing new software.

The libraries or databases used in SimaPro for the LCA analysis were as follows:

- ecoinvent 3: Allocation at point of substitution—unit
- ecoinvent 3: Allocation, cutoff by classification—unit
- Agri-footprint: Mass allocation

The database ecoinvent contains a large variety of data useful for building an LCI and is made up of more than 10,000 process-based interlinked databases about energy generation, agriculture, construction, infrastructure production of materials, chemicals, fuels, etc., while the database agri-footprint contains a large variety of LCI data about fertilizers, crops, agricultural operations, food, and crop and animal product processing [72]. Both databases were used to build the LCI. The environmental impact assessment method chosen was ILCD 2011 Midpoint+, EU27 2010, equal weighting, which is a midpoint method released in 2012 by the European Commission. It supports the use of those characterization factors recommended by the ILCD and based on well-established environmental impact assessment categories, factors, and models [72]. The calculation of a single score was conducted in accordance with the guidelines from the European Commission [73] and Pizzol et al. [74]. The unit points (Pt) express the total environmental impact presented as a single score in which characterization, normalization, and weighting are combined and taken into account [72].

The other building block of the combination carried out in this article was LCA of FOX apple juice with reference to conventional apple juice. The technology applied to produce FOX apple juice was explained by Zdravkovic et al. [42]. Different from our approach, the conventional system Zdravkovic et al. described was small-scale apple juice production, while in this study, the conventional system was built to describe a production on a large scale. The information used for the reference product, conventional apple juice, is summarized in the following paragraph. The data regarding the apples used in juice production were mostly retrieved from the life cycle assessment of organic and conventional apple supply chains in the north of Italy [75], while those regarding the manufacturing of juice were from the report “Harmonised Environmental Sustainability in the European food and drink chain” [76]. Such apples are harvested, sorted, and sent to the manufacturing plant where the fruit juice is extracted. The fruits are processed through a press, where separation between the pulp and juice occurs. After this, the juice is thermally treated through a pasteurization procedure and finally stored under controlled conditions [76]. The conventionally produced and treated apple juice is then sent through the bottling phase, in which glass bottles are filled, re-pasteurized, capped, and labeled in a safe environment in order to ensure the safety and asepticity of the apple juice [76]. The obtained product goes through secondary and tertiary packaging procedures in order to be distributed to retailers. The data regarding secondary and tertiary packaging were taken from the article “Proposal of Package-to-Product Indicator for Carbon Footprint Assessment with Focus on the Czech Republic” [77]. Data from transportation were also retrieved from the article “energy balance for locally grown versus imported apple fruit” [78]. Once at the retailers [79, 80], conventional apple juice is stored and then sold to customers [81, 82]. Data for waste management were retrieved from the Federal Ministry for the Environment, Nature Conservation, and Nuclear Safety (BMU) [83].

### The three scenarios

The scenario method was a widely used instrument in foresight that assists the user in dealing with uncertainties. The scenarios discover the future by identifying different future aspects, thus providing a basis for decision-making. Moreover, working with scenarios, decision-makers can gain awareness of different, possible future developments, uncertainties in the environment of the studies system, and signs of discontinuities. Scenarios are based on different future projections of the key factors , and they mostly include a wide range of qualitative descriptions, presented as story lines about alternative futures [84–87]. There are many different approaches in scenario development; however, they usually start by deconstructing a system into a set of individual key factors, for which different future projections are defined. Finally, these assumptions are combined into different scenarios. Spaniol and Rowland [88] outlined that “scenarios should be possible and plausible while taking the proper form of a story or narrative description, and that scenarios exist in sets that are systematically prepared to coexist as meaningfully alternatives to one another.” Scenarios are constructed with different time horizons. In many processes, static considerations, as in our scenario analysis, are made from a point in time in the future—these are referred to as static scenarios or end-state scenarios. Alternatively, the dynamics of the development over several time steps in the future are considered, e.g., dynamic or sequential scenarios or chain scenarios. As an example for the dynamic construction of scenarios, the deterministic-dynamic concept of Kane [89] is well qualified. Kane’s simulation was originally developed to derive quantitative statements about dynamic processes from qualitative information. Due to the similar objectives of his method and cross-impact analysis, this approach is suitable for investigating the mutual influences of different events and is therefore used in the context of scenario analysis [90].

In this paper, we provide snapshots of these three scenarios for Europe’s food sector in 2035, which are mentioned in Fig. 2. Details about the development and descriptions of these scenarios, the key factors, and the assumptions can be found in the work of Moller et al. [36]. Scenario A, “policy secures sustainability,” describes a world where agriculture is increasingly nationalized, in which the state owns large parts of the agricultural area and takes care of the well-being of the citizens but only to the extent that their basic needs are satisfied. There is no need for the consumer to understand the complexity of the agri-food value chain. Sustainability is not prominent in purchasing decisions and is only promoted through tax reliefs. New forms of food production have taken hold, and new technologies, above all, are used to better control the complex value chain. In scenario B, “society drives sustainability,” on the one hand, agricultural land is owned by many different stakeholders, and on the other hand, agricultural production is supplemented by urban farming taking place in a 1-mile radius of consumption. Local biodiversity is appreciated and taken care of, while the import of exotic foods is largely avoided. High food prices are accepted if sustainable production covers all three dimensions of sustainability. In conclusion, this means that sustainable behavior is established in the middle of society. At the same time, society is wide open to all forms of new technologies in the agri-food sector. In contrast, in scenario C, “CO_2_-currency and retailers dominate trade and consumption,” big players are dominant in a globalized world. As global markets are still in place, consumers can enjoy a great variety of foods, and the flexibility of consumption choice is the main criterion to buy. Large retailers and sales groups have data sovereignty and therewith control of complex agri-food value chains. Technological progress is the main means to overcome global challenges. On the one hand, climate protection takes place because there is the necessity to do so, and on the other hand, agriculture becomes increasingly intensified with negative consequences for biodiversity.

### The combination of the three scenarios and the LCA of FOX apple juice

In this study, a concept to combine future scenarios with LCA was developed, in line with van der Giesen et al.’s asking to “develop or expand on existing LCI databases like ecoinvent to represent potential future situations via clearly defined scenarios” [18]. Scenarios of the future European food sector [36] were used to test the conceptual framework of quantifying the qualitative descriptions and their subsequent combination with LCA. The qualitative description was translated to a change, in percentage, the parameters of the life cycle inventory (F) coupled with an explanation of the reason for the said change. We wanted to adjust the LCI in a way that it is “descriptive of future technological developments and accounting for parameter and scenario uncertainty in exploring how the life cycle inventory may change with future developments” [32]. On the one hand, the scenarios themselves were developed with experts, and on the other hand, the changes in the LCI were also supported by expert judgment.

In the current study, qualitative scenarios were used and translated into quantitative values, which were fed into the background system of the LCA. With this, a background system for each of the scenarios was created. Consequently, a foreground system can be combined with the different background systems [91]. The scenarios and the data from the LCI were elaborated in the project FOX [36, 42]. The results of this calculation showed what the product under consideration would look like in the different future worlds and what environmental consequences would be connected with it.

It should be noted that the LCA in this study followed an attributional approach [91] and therefore “assesses the impact of realisation of the functional unit and does not consider (environmental) impacts on the overall economy if the product or service under assessment would replace the current situation” [92]. In addition, it has to be highlighted that we refer to the combination of LCA and qualitative scenarios as future-oriented LCA. Furthermore, the foreground system was not adapted, because we assumed no change in the technology itself. The changes looked at were assumed to happen in the environment of the technology in the respective different futures. Hence, the manipulation was conducted in the background system of the life cycle inventory. The changes that occur in the development of a technology were not the topic of this article; instead, a detailed review of prospective LCA studies focusing on technology development and its integration in future-oriented LCA can be found in the work of Arvidsson et al. [46]. In contrast to this work that omitted a changing background system, we focused on changes in the environment of the technology. The results can thus be fed back to technology developers to see how robust the technology appears under different future framework conditions. In addition, we wanted to define the usefulness of the application of qualitative scenarios in prospective LCA.

The actual integration of the qualitative scenarios was achieved via two steps: First, the inventory parameters were adapted using information from the scenarios; second, the adapted inventory was used to calculate the prospective LCA results. This proceeding is in line with the work of Mendoza Beltran et al. [34], although they used scenarios from an integrated assessment model. The following parameters evolving from the LCI were used for the expert assessment (see Table 1).

**Table 1:** Parameters from the life cycle inventory (LCI) used for the quantification of the three scenarios

	Supply chain stage	Parameter
1	Production	NPK mineral fertilizer 15-15-15
2		Pesticide, unspecified
3		Diesel
4		Water, unspecified origin
5		Electricity
6		Transport 1—on the farm
7	Processing	Transport 2—from farm to processing plant
8		Wooden pallet
9		Compressed air
10		Packaging film, low-density polyethylene (second and third packaging)
11		Corrugated board box (second packaging)
12		HDPE bottle E (thermoplastic)
13		Tap water
14		Electricity
15	Retail	Refrigerant
16		Electricity
17		Tap water
18	Consumption	Electricity
19		LDPE (low-density polyethylene)
20		HDPE (high-density polyethylene)
21	Disposal	Waste collection
22		Dumping
23		Incineration
24		Biological treatment (compost)
25		Biogas

In order to obtain the respective values for modeling the background system toward different futures, scientific articles, forecasts of authorities, and expert interviews were used. Thonemann et al. [33] proposed to use, inter alia, scientific articles and expert interviews to model the foreground system. Expert opinion can be used to encounter highly uncertain future developments by assuming that the world cannot be fully known, but rather, different parts of existing knowledge have to be combined [18]. This is supported by the involvement of experts from different disciplines (experts in the fields of agriculture, mobility, infrastructure, energy, and waste management). The experts indicated the expected change of a certain parameter in the three scenarios in percentages. These numbers were taken as a coefficient, with 1 being 100%.

As some decisions in the assessment of future developments cannot be based on natural science only, assumptions on the behavior of the actors influencing the parameters of the background system were based on expert assessment [20, 91]. The selected scenarios present future situations for all of the parameters of LCA [34]. The parameters shown in Table 1 were clustered into fields of expertise (agricultural production, fuel and transport, water, energy, packaging, and disposal). For each of these topics, interviews with one to three experts were conducted. The interviews were held from July to September 2021. After introducing the scenarios and the context of the project FOX, the experts assessed the influence of the framework conditions explained by the scenario narrative on the development of the parameters in the future. For example, an expert on packaging machines and materials assessed the use of different packing materials in the three scenarios. The framework conditions in each scenario led to a relative increase or decrease in used packaging materials compared to the current situation. The adapted values of the parameters were then used for the calculation of the future-oriented LCA.

### Difference between conventional and FOX apple juice production in the LCI

In the LCA, comparing FOX and conventional apple juice, some changes were implemented in the LCI. They are summarized below, structured along the stages of the supply chain and highlighting the key processes of FOX versus conventional technology.

In the *production stage*, the agricultural data concerning the life cycle inventory (fertilizers, pesticides, water, energy, fuel, and operations in the field) are not different compared to conventional apple juice production, because it is assumed that the same apples are being processed. The underlying assumption is that most farmers do not change their agricultural practices in the short-term, even if they decide to process their apples with FOX technology and their production decisions are based more on other framework conditions [93]. Therefore, we supposed that agricultural production will be the same for FOX and conventional apple juice.

The parameter most affected by the adoption of FOX technology is *transport*. This is due to the adoption of small-scale flexible and mobile processing units aimed at shortening the food supply chain of fruits and vegetables in the European Union [94]. Transport is a difficult-to-quantify parameter and translates into an LCA inventory, because it experiences a large variety in terms of both modes of transport (air, water, roads, and railways) and means of transport (cars, tractors, trains, planes, ships, etc.) and because it is greatly affected by space and time variations (every country has its own transport infrastructure, which varies year after year). A lot of research has been conducted to quantify both the environmental and economic costs associated with transportation [95]. Due to the complex nature of transport, these studies are often difficult to apply for different contexts, and regional considerations cannot be transferred without adapting to local conditions. Very comprehensive LCA studies modeling transport focuses on the mobility sector itself, for example, the design of a sustainable public transportation system [96]. Other studies look at specific transportation steps, e.g., connected to agricultural production [75], whereas many others just ignore transportation because of a lack of data and complexity [97].

In the *processing* stage, one aspect has to be taken into consideration: The machinery to process apples is different for the FOX system. In the FOX approach, as new technologies are used, they have different energy, fuel, and water requirements [42], which result in consistent quantitative differences in the LCI data in the processing stage, although the overall impact of the building materials (steel, electronics, and plastic) appears to be negligible and they are considered to represent another difference in the LCI data between the conventional and FOX approaches. Transport from the processing plant to the retailers is supposed to be shortened.

The two penultimate stages are *retail* and *consumption*. Apart from transportation, the impacts derived from retailers are assumed to be those derived from electricity use, tertiary packaging, and refrigerant leaks, while the impacts related to consumers, again apart from transportation, are those derived from electricity use, refrigerant leaks, and water used for cleaning dishes. There is no evidence to affirm that water, electricity, or refrigerant use is different between the conventional and FOX approaches, although for a future scenario, all of these parameters may vary. In the retail, consumption, and disposal stages, transport is supposed to be shortened.

For the last stage, the *disposal*, there is no evidence to affirm that waste collection and treatment will be different between the conventional and the FOX approach, although for a future scenario all of these parameters may vary.

## Results

This section shows the calculated changes in environmental impact based on the changes determined in the inventory. As explained, the qualitative scenarios were translated into quantitative changes of the parameters of the LCI based on expert assessment. The adapted inventory was then used to calculate the prospective LCA results, shown as changes in the impact assessment of FOX and conventional apple juice for the present and the three future scenarios, respectively. In other words, the first output of the combination adapted ecoinvent databases that are scenario dependent. This proceeding is in line with Mendoza Beltran et al. [34], although they used scenarios from an integrated assessment model. Bisinella et al. described the approach of integrating externally developed scenarios into LCA with specific methodological variations in their review study [50]. The life cycle impact assessment methodology was applied in accordance with the *International Reference for Life Cycle Data System* handbook by the European Commission [73]. Emissions and resources for the studied product compiled in the LCI were “assigned to the corresponding impact categories and [...] converted into quantitative impact indicators using characterization factors” [98].

Table 2 shows that the environmental impact of the FOX apple juice was lower compared to that of the conventional apple juice in most of the impact categories in the present and future situations. Comparing the scenarios for either the FOX or conventional apple juice, the “society” scenario proved to have the lowest impact in most of the impact categories.

**Table 2:** Environmental impacts of FOX^a^ and conventional apple juice calculated with ILCD 2011 Midpoint+ V1.11

Midpoint impact category	Unit	FOX apple juice, present scenario	FOX apple juice, “policy” scenario	FOX apple juice, “society” scenario	FOX apple juice, “retail” scenario	Conventional apple juice, present scenario	Conventional apple juice, “policy” scenario	Conventional apple juice, “society” scenario	Conventional apple juice, “retail” scenario
Climate change	kg CO_2_ eq	2.98	1.30	1.25	1.36	3.50	1.52	1.42	1.56
Ozone depletion	kg CFC-11 eq	9.83·10^−7^	6.33·10^−7^	5.91·10^−7^	6.86·10^−7^	9.10·10^−7^	5.08·10^−7^	4.56·10^−7^	5.46·10^−7^
Human toxicity, noncancer effects	CTUh	9.47·10^−7^	2.69·10^−7^	2.42·10^−7^	3.05·10^−7^	1.15·10^−7^	3.43·10^−7^	3.06·10^−7^	3.69·10^−7^
Human toxicity, cancer effects	CTUh	2.68·10^−7^	7.23·10^−8^	6.40·10^−8^	8.16·10^−8^	3.24·10^−7^	9.07·10^−8^	7.99·10^−8^	9.75·10^−8^
Particulate matter	kg PM_2.5_ eq	7.26·10^−4^	5.56·10^−4^	5.07·10^−4^	5.51·10^−4^	8.03·10^−4^	6.23·10^−4^	5.91·10^−4^	6.06·10^−4^
Ionizing radiation HH	kBq U235 eq	5.08·10^−1^	3.95·10^−2^	3.52·10^−2^	4.65·10^−2^	6.12·10^−1^	4.68·10^−2^	4.13·10^−2^	5.17·10^−2^
Ionizing radiation E (interim)	CTUe	1.10·10^−6^	1.87·10^−7^	1.61·10^−7^	2.29·10^−7^	1.30·10^−6^	2.14·10^−7^	1.83·10^−7^	2.47·10^−7^
Photochemical ozone formation	kg NMVOC eq	5.07·10^−3^	3.05·10^−3^	2.96·10^−3^	3.61·10^−3^	6.10·10^−3^	3.79·10^−3^	3.32·10^−3^	4.25·10^−3^
Acidification	molc H+ eq	1.77·10^−2^	1.43·10^−2^	1.34·10^−2^	1.23·10^−2^	1.90·10^−2^	1.52·10^−2^	1.56·10^−2^	1.31·10^−2^
Terrestrial eutrophication	molc N eq	6.46·10^−2^	5.65·10^−2^	5.36·10^−2^	4.65·10^−2^	6.85·10^−2^	5.96·10^−2^	6.29·10^−2^	4.92·10^−2^
Freshwater eutrophication	kg P eq	3.39·10^−3^	2.89·10^−4^	2.59·10^−4^	3.29·10^−4^	4.11·10^−3^	3.64·10^−4^	3.24·10^−4^	3.92·10^−4^
Marine eutrophication	kg N eq	2.72·10^−3^	1.37·10^−3^	1.31·10^−3^	1.47·10^−3^	3.22·10^−3^	1.62·10^−3^	1.49·10^−3^	1.69·10^−3^
Freshwater ecotoxicity	CTUe	2.93·10^−1^	1.29·10^−1^	1.16·10^−1^	1.47·10^−1^	3.54·10^−1^	1.64·10^−1^	1.46·10^−1^	1.76·10^−1^
Land use	kg C deficit	8.24	1.16·10^−1^	1.04·10^−1^	1.30·10^−1^	9.89	1.45·10^−1^	1.29·10^−1^	1.56·10^−1^
Water resource depletion	m^3^ water eq	1.03·10^−1^	8.27·10^−2^	7.24·10^−2^	9.29·10^−2^	6.94·10^−2^	5.57·10^−2^	4.91·10^−2^	6.25·10^−2^
Mineral, fossil and renewable resource depletion	kg Sb eq	8.41·10^−5^	1.05·10^−4^	9.50·10^−5^	1.20·10^−4^	9.76·10^−5^	1.31·10^−4^	1.16·10^−4^	1.40·10^−4^

^a^Food processing in a boX, European Union’s Horizon 2020 research and innovation program

**Fig. 3:**
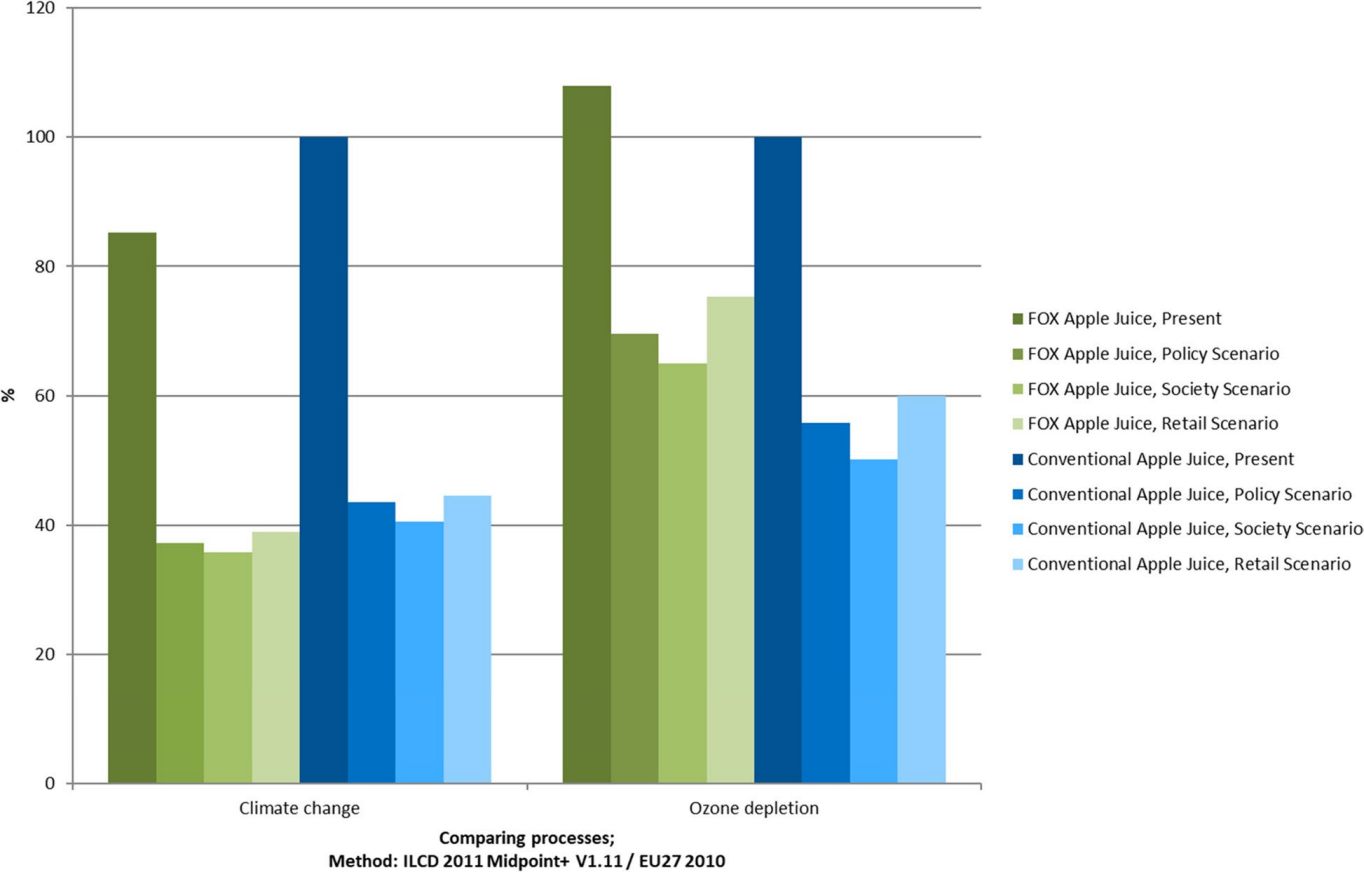
Midpoint categories of FOX and conventional apple juice for the present and three future scenarios, respectively; characterized factors, method: ILCD 2011 Midpoint+ V1.11

Figure 3 shows two out of the 16 impact categories for the present situation and the three future scenarios for the FOX and conventional apple juice, respectively. When comparing all of the impact categories, climate change and ozone depletion showed the same trend as approximately two-thirds of the categories. The present situation shows a higher impact than the three future situations. Meanwhile, with climate change, the FOX apple juice had less of an impact than the conventional apple juice, while for ozone depletion, it was the other way around. Ozone depletion is mostly influenced by electricity production, as well as the production of refrigerant. For the FOX apple juice, the need for cooling is higher, which is reflected in the greater impact in this category.

**Fig. 4:**
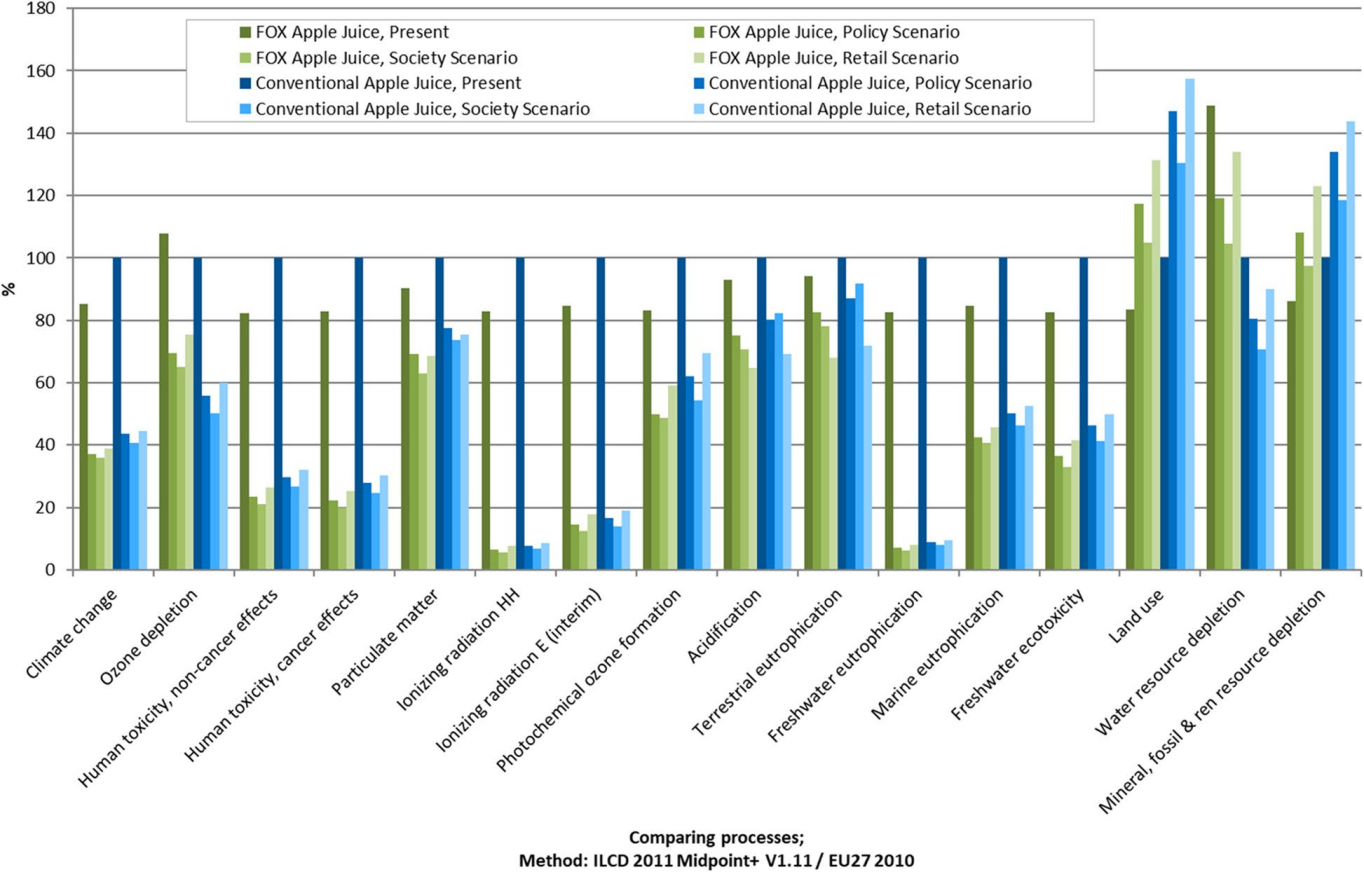
Relative contributions to the environmental impact with the conventional apple juice in the present as the reference value at 100%; characterized factors, method: ILCD 2011 Midpoint+ V1.11

For the impact categories land use, water resource depletion, and mineral, fossil, and renewable resource depletion (see Fig. 4), the future scenarios showed a higher impact than the present situation. The midpoint category land use was defined in the technical guidelines on the “Characterisation factors of the ILCD Recommended Life Cycle Impact Assessment methods" by the Joint Research Centre as the deficit of soil organic matter (SOM) and quantified with the flow property of the impact indicator "mass deficit of soil organic carbo” in the reference unit kilograms [98, 99]. Further effects of the changes in SOM on biodiversity and soil quality are not discussed in this work. For land use change, as well as for mineral, fossil, and renewable resource depletion, conventional apple juice in the future showed an especially high impact compared to water resource depletion. Therefore, the data showed that FOX technology has a high impact in these three categories. Looking at the present situation, the FOX apple juice had a high impact on water resource depletion. Meanwhile, looking at the stage-wise results, water resource depletion was mostly triggered by agricultural production (see Fig. 6).

For land use, as well as for mineral, fossil, and renewable resource depletion, the present situation for FOX apple juice showed less impact than for the three future scenarios. For land use, this could be due to the fact that we supposed less pesticide and fertilizer use in the future, and therefore, more arable land will be needed to produce the same amount of food. The conventional apple juice showed this trend as well but with an even higher impact. This could be attributed to the fact that no difference in the agricultural stage between FOX and conventional apple juice was assumed for future apple production, and that the impacts of other stages of the supply chain add to the higher impacts for the conventional juice in these two categories.

**Fig. 5:**
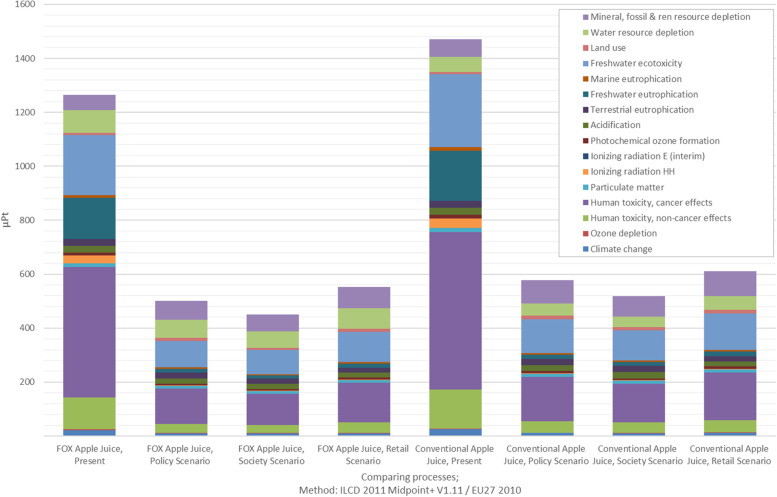
Results of the impact assessment presented as a single score for the FOX and conventional apple juice in the present and three future scenarios, respectively; weighted factors, method: ILCD 2011 Midpoint+ V1.11

The results of the single score are shown in Fig. 5. The first four columns, starting from the left, refer to the FOX apple juice in the present scenario and in the three future scenarios, respectively. Meanwhile, the last four columns refer to the conventional apple juice in the present scenario and in the three future scenarios, respectively. The different colors in each column represent a different environmental impact category, e.g., climate change, land use, or acidification. All of the different impact categories were combined into a sort of aggregated environmental global score, which, by taking a weighted average of all the different impact categories, attempted to quantify—in a simple and direct way—the overall environmental impact of the products considered for the LCA analysis. “The weighting determines how severely the environmental impacts of one category are assessed compared to the impacts of other categories” [100]. The normalized results of the life cycle impact assessment (LCIA) “can be multiplied by a set of weighting factors, that indicate the different relevance that the different impact categories” [73]. Frischknecht and Büsser Knöpfel [100] stated that weighting should be understood as a “dimensionless quantity determined exclusively by the ratio of current to critical flow”, meaning that the absolute levels of flow will not influence the weighting. In the current study, an equal weighting was applied, in contrast to other weighting schemes, which derived the values for the weights from expert panels or public surveys [101]. The most favorable scenario for the environment across the impact categories was the scenario “society.” In general, all future scenarios showed a lower environmental impact, which goes along with the fact that all of them are supposed to reach sustainability goals. The main contributors to environmental single score impacts were human toxicity (cancer effects), freshwater ecotoxicity, and mineral, fossil, and renewable resource depletion with unchanged importance in the future as well. Meanwhile, freshwater eutrophication and water resource depletion had a higher impact in the present than in the future.

The environmental benefits observed in all three scenarios are related to the fact that the developed scenarios claimed to be more environmentally friendly than the present situation. However, it was not specified which degree of sustainability has to be achieved, nor by which means. As shown in Table 3, the FOX apple juice analyzed in the present scored 1264.16 μPt^4^. The same juice scored 500.49 μPt in the scenario “policy,” 449.90 μPt in the scenario “society,” and 552.06 μPt in the scenario “retail,” representing a reduction in the environmental impact of 60.41% (“policy” scenario), 64.41% (“society” scenario), and 56.33% (“retail” scenario), respectively. Similarly, conventional apple juice scored 1470.54 μPt in the present, 578.29 μPt in the scenario “policy,” 519.65 μPt in the scenario “society,” and 611.57 in the scenario “retail,” indicating a reduction in the environmental impact of 60.67% (“policy” scenario), 64.66% (“society” scenario), and 58.41% (“retail” scenario), respectively. It is apparent that no matter which future we are heading to, the future itself, if we assume it is a sustainable one, will make the biggest difference in reducing the environmental impact, because we assume reductions in one or another resource in the future.

**Table 3:** Total value for the single score for all of the alternatives

Unit	FOX apple juice present scenario	FOX apple juice “policy” scenario	FOX apple juice, “society” scenario	FOX apple juice, “retail” scenario	Conventional apple juice, present scenario	Conventional apple juice, “policy” scenario	Conventional apple juice, “society” scenario	Conventional apple juice, “retail” scenario
μPt	1264.16	500.49	449.90	552.06	1470.54	578.29	519.65	611.57

Looking at Figure 5 and Table 3, it is evident that in all of the analyzed situations (present and the three future scenarios), FOX apple juice appeared to be the more environmentally favorable choice when compared to the conventional non-PEF-treated juice. The reduction in environmental impact due to the technology can be seen in the scenario "society," where FOX apple juice with 449.90 μPt had a 13.42% reduction in environmental impact compared to conventional apple juice in the same scenario, with 519.65 μPt. In the scenario "policy," the reduction was 13.45%, and in the scenario "retail," 9.73%.

**Fig. 6:**
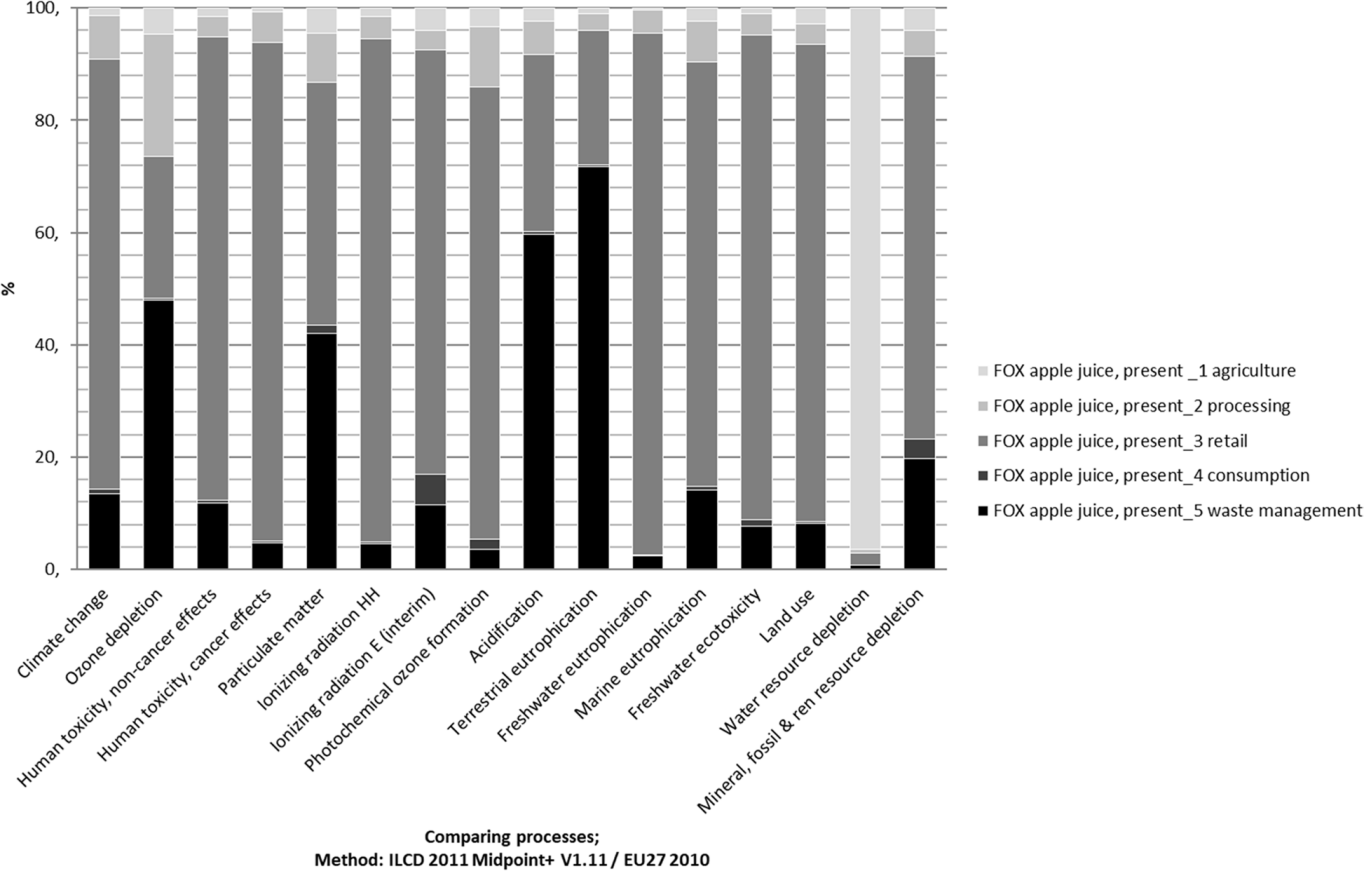
Impact per supply chain stage of FOX apple juice in the present; characterized factors, method: ILCD 2011 Midpoint+ V1.11

To better understand the importance of each of the stages of the supply chain, the disaggregated impact is presented in Figure 6. The columns show the respective share of impact of each stage of the supply chain. For most of the midpoint categories, the retail stage had the highest proportional share of the impact, followed by the waste management stage. For water resource depletion, the agricultural stage was the most impactful, which was confirmed by Frankowska et al. [102]. Logistics is included in all the relevant stages, farming logistics in the agricultural stage, from farm to processing in the processing stage, and from processing to retail, within the retail stage to the retail stage, and from retail to consumer in the consumption stage.

## Discussion

Quantitative forecasting methods cannot adequately capture qualitative factors such as political, social, ecological, and technological developments. In most cases, structural discontinuities cannot be taken into account either, since mathematical-statistical models assume that patterns observed in the past will continue to be valid in the future [103]. An important feature of qualitative forecasting methods is the collection of often nonquantifiable expert knowledge, including subjective opinions, which are collected in expert-based approaches. The focus is less on explaining and more on understanding [104]. However, purely qualitative statements about the future are often difficult to communicate as a basis for decision-making.

The combination of quantitative and qualitative methods can uncover advantages of both areas of research [105, 106], as well as contribute to a more comprehensive picture of the studied system or topic [107–109]. Different options of combining qualitative information exist, from foresight studies with quantitative research—for example, the story and simulation approach translates stakeholder-based narratives into quantitative parameters [110, 111]. The aim of the present work was to show how qualitative scenarios can be linked to quantitative LCA. The exact point of the combination of the qualitative framework conditions, the scenarios, and the quantitative assessment was the LCI parameters in the inventory. This was done in order to show the effects of future framework scenarios on the technology studied within the LCA approach, showing different impacts of the technology depending on the surroundings in which it operates. LCA, on the one hand, is a methodology used to assess processes or products under present conditions; however, for decision-making and long-term investment cycles, how the environment will evolve in the future and assessing the impacts of these changes on the studied system are highly relevant.

LCA and other quantitative methods can be combined with quantitative studies to show a broader picture of their impact. In addition, qualitative scenarios can contribute to make quantitative models future-proof. The combination can be applied to any kind of LCA in the proposed way, provided that qualitative scenarios for the respective system under investigation are available or can be developed. Furthermore, research projects in which quantitative and qualitative methods are applied should be encouraged to look for methodological combinations. As described in “State of the art—future-oriented life cycle assessment,” there are different points for the integration, and regarding the aim and availability of preliminary work, a suitable option can be defined.

In this article, qualitative scenarios were combined with LCA through the inventory stage (LCI). Considering the many assumptions that had to be taken to model the background system, the results should be interpreted as “a possible implication a technology can have under a specific set of assumptions” [18]. These results can be used to initiate debate about the future potential of the respective technology. Therefore, in line with Olsen et al. [32], we wanted to provide a basis for “current strategic discussion and decision-making [...] rather than predicting precisely.” Villares et al. [45] concluded that outcomes of the ex ante application of LCA should not be communicated as the final result but rather point to the environmental hotspots and therewith have an indicating purpose. In general, the many assumptions and decisions on the integration or exclusion of aspects, stages, or impacts, LCA can still be a valuable tool to indicate hotspots of environmental burden [52].

The values from the impact assessment confirmed the environmental benefits from FOX technology. All three future scenarios related to FOX apple juice appeared to be less impactful under an environmental point of view when compared to conventional apple juice. Of course, an intervention targeting technological improvements is only one way to address environmental burden. Changing framework conditions, influenced by multiple sources, such as policies, can translate into a higher change of impact. Nevertheless, it has to be pointed out that the results show possibilities of future potential of the respective technology in alternative futures. However, the conditions of the framework scenarios are just possible developments, and even more specifically in the case of the scenarios used in this study, they describe futures in which sustainability is followed in one way or another. Therefore, the decision regarding which scenarios are to be used for the combination has to consider the scope of the discussion that should be triggered. If explorative scenarios are chosen for the combination, the results could show an even broader picture of the technology under future framework conditions. The most remarkable observation that emerged from the data comparison was that for both FOX and conventional apple juice, the scenario “society” was the least impactful from an environmental point of view, followed by the scenario “policy” and last the scenario “retail.” This is associated with the single assumptions forming the scenario; as for the scenario “society,” it was assumed that the majority of the population maintains a sustainable lifestyle that allows the full potential of FOX technology to be realized. For the individual parameters assessed, the expert judgment did not always see the highest reduction in inputs in the scenario “society.” For example, concerning the secondary and tertiary packaging scenario “policy,” the highest reduction (80%) was seen because sustainability goals are strictly followed, resulting in declining convenience, including for the consumer. Meanwhile, in the scenario “society,” the reduction (50%) was driven by an increase in local consumption, making secondary and tertiary packaging redundant in some circumstance, but with less success to avoid than in the scenario “policy.” Due to the unchanged consumption patterns, the reduction was small in the scenario “retail” (20%). The sole driver here was the necessity to reach sustainability goals for the companies, which did not have the same impact as the framework conditions in the other two scenarios. The expert assessment was accordingly conducted for a further 25 parameters.

Basset-Mens et al. studied the environmental impacts of imported and locally grown fruits for the French market in a cradle-to-farm-gate LCA study [112] and stated that “fruit cropping systems have seldom been studied using life cycle assessment,” like was done in this work. Furthermore, in the current study, no difference was supposed between the FOX and conventional apple juice for the agricultural production stage. However, as FOX technology was designed to operate at a small scale, the higher importance of locally grown fruits can be taken into account. When further studying the technology, a differentiation should be made between the various sources of apples used for FOX and conventional technologies. Furthermore, the explicit results for the supply chain showed that much of the environmental impact is caused by retail and waste management, implying that these are significant levers for a reduction in impact. This is opposed to Dijkman et al. [52], as they stated that “studies showed that in the full life cycle of a food product, it is often the farm inputs and agricultural stages where most environmental impacts arise.” Of course, this observation is highly dependent on the produced product; for example, animal husbandry has a high impact on global warming or acidification, and eutrophication impacts are important in fertilizer-intense species [52]. Even though the leverage seems to be very low at certain stages, implying that the impact of an intervention is lower at that stage, future frameworks could change this ratio. For example, in a future where local food circles prevail, the impact of the processing technology will have more importance than in a future where international trade and big retailers are dominant. Therefore, comparative analysis along the supply chain can provide many insights into current and future hotspots for intervention.

Concerning the expert assessment of changes in the various parameters, some difficulties can be noted. “Projections concerning fertilizer use are difficult for various reasons” [63]; for example, agricultural management or soil quality can influence the efficiency of fertilizer use to a great extent. This influenced the expert assessment on this parameter. In this sense, Odegard and van der Voet [63] pointed out that if the quantification of assumptions is changed, then the results will also change. They further stated that “scenarios [..] are not made to assess what will happen, but what could happen, given such a set of consistent assumptions” [63]. This means that the modeled results should not be understood as exact values but rather as presenting a range of possibilities. Another challenge for the expert assessment and, therefore, the translation of qualitative assumptions into quantitative values is the understanding and capturing of the scenario itself. For the generation of future values, it is important that first, scenarios are well explained and detailed enough to make it possible for experts to assess the very specific parameters of the LCA, and second, the accessibility of experts on different thematic fields has to be ensured. Where expert assessment is not retrievable or is too time-consuming, the values for parameters can be searched for in literature. A challenge here is that the searched values have to meet the point in time set for the scenarios. Different sources probably have to be consulted to find time-specific values for various parameters. In additions, values are needed for not only one scenario but for several, and furthermore, the retrieved values have to meet the described scenario narrative. As mentioned before, this leads to the conclusion that the herewith-generated results should only be used to trigger discussion and can be seen as a tool for engaging in a future-oriented thinking within the present value-based arena of LCA or other quantitative methods. Going back into the thinking of preciseness, often communicated with quantitative assessments, should be avoided.

The further we go into the future, the more difficult it is to make assumptions on the values to feed into the background system, since more uncertainty is connected to these values [46]. Uncertainty can be addressed “by means of relevant and consistent scenarios representing possible futures” [34]. Van der Giesen et al. postulated that the quantification of uncertainties in the future would only entail another dimension of uncertainty [18]. In addition, a difficulty for the experts was working with already existing, spelled-out qualitative descriptions of alternative futures when assessing the development of the different parameters in the year 2035. With our methodology of developing consistent and plausible qualitative scenarios, as explained by Moller et al. [36], in a transparent approach, we aimed at a broad stakeholder involvement and acceptance. Therefore, we worked with already existing complete descriptions of alternative framework scenarios and assigned the respective values for the parameters according to the different scenarios, in contrast to modeling scenarios based on quantitative data only [34]. Niero et al. [61] worked with focus points, as “the main deviations from the baseline scenario.” The variation in the values for these focus points or parameters leads to the formation of scenarios, as indicated by different authors [61, 113].

Furthermore, in the current study, the entire life cycle of a product was considered, as recommended in the Norm ISO 14040 [20]. On the one hand, this ensured that no stage of the supply chain with a big environmental burden was overlooked, but on the other hand, a focus on one stage could save resources in conducting research and might be the better option depending on the research question. For example, Hospido et al. [114] decided to exclude the downstream stages retailing, consumption, and waste management, arguing that they wanted to assess the differences in the production, processing, and transportation of lettuce produced under different conditions. As the current study connected the holistic framework scenarios for the year 2035 with the LCA of FOX and conventional apple juice, it was more suitable to include the whole supply chain to obtain or stick to the broader picture.

Future economic and societal changes were captured through the scenarios and expressed in different key factors, e.g., growth paradigm in change, purchasing behavior related to food, public and private investment in food and agriculture, society’s attitude toward new technologies, and appreciation of products promoting ecosystem services. This is in line with the macro-LCA approaches by Dandres et al. [115], combining “LCA with future changes in economic structure and energy production” [34].

### Limitations

As already mentioned in the methodology and despite the fact that other studies have postulated the foreground system to be most crucial, we focused on modeling the background system [33]. This is because the developed technology is not meant to be scaled up in the future, but the framework conditions under which it operates change over time. This focus on adapting the background system can be seen as a modeling choice, rather than a limitation of the approach [34]. Furthermore, it has to be stated that the background system in general is of high importance, as “background processes usually make up to 99% of all unit processes in a product system and only occasionally below 95%” [18].

To overcome the general limitation of the completeness of data and equal accuracy for all of the processes within LCA, Olsen et al. [32] proposed to understand LCA as “a process of learning in which the people and organizations involved in the process acquire knowledge about the product and its environmental impacts, the limitations will often appear less severe.” We conclude that the applied method in this study—where future values are also brought into the assessment—addresses the need of continuously adjusting the LCA according to the changes in the environment and therefore captures more severe influences compared to some detailed adjustment within the present LCA. The foreseeable change of just one important parameter in the near future may make it useful to adjust the LCA accordingly, to calculate with different possible values, and to critically review the results.

The present study only investigated the impact of future developments on the environmental dimension of sustainability. Taking into consideration the broader impacts on the economy and society that a new technology entails, a comprehensive analysis should include trade-offs between the three dimensions of sustainability in the future. Therefore, the results in the first instance show the applicability of the method to combine future scenarios with impact assessment by the example of LCA. In this sense, studies on the environmental impact of agricultural production [52, 59, 116] could, on the one hand, be expanded to social and economic impacts and, on the other hand, also take future developments into account. One option to combine the economic perspective and a future-oriented assessment is the estimation of the potential market share of an innovation, among other indicators [18]. Nevertheless, it has to be stated that the assessment of the role of regulating policies and economic measures, respectively, was indirectly conducted through assumptions within the different scenarios. Socioeconomic trends have a great potential to influence the acceptance of a product or a technology [34, 47]. Therefore, this approach enabled the environmental assessment of a product using LCA to not only be put into the future context but to see the influence of economic and political changes in the framework of the technology in question as well.

Due to data availability, country-specific values were not always retrievable for the assessment of future developments, nor for the current values within the ecoinvent database. For parameters with high impact within the LCIA, country-specific values can make a huge difference. For example, electricity was defined in the sensitivity analysis of the LCA on the new technology to produce apple juice conducted in the project FOX that contributes to a high share of the total impact of the product [42]. In addition, studies have called for the integration of biodiversity and ecosystem services in LCIA [117–120] and for applying an approach that accounts for the importance of their spatial heterogeneity [121, 122]. Future studies integrating these impact categories should therefore be aware of the importance of spatial modeling.

Expert opinions are based on the individual background of the different professionals. Therefore, we recommend for future studies to include experts from different stakeholder groups in the assessment of specific parameters to capture the full range of possible variation in values. In order to select a value, if more than one value for a certain parameter in the respective alternative future can be identified, the authors of this study indicate preference of the value from the expert assessment. This is in line with Thonemann et al. [33], who stated that for “the LCI, primary data like expert interviews should be first used, and if no primary data is accessible, secondary or proxy data, e.g., from the literature, should be used.” Furthermore, Buyle et al. [47] explained that, on the one hand, the future is sometimes too complex for assessment with rationale models alone, but, on the other hand, the validation of the assessment and its objectivity is potential disadvantages of participatory methods.

As the focus of the study was on testing the methodological approach of combining qualitative scenarios with LCA, it is not inconceivable that a more comprehensive search for datasets describing changes in the future composition of input factors, such as the integration of biodiesel, biopesticides, or bioplastics, would have led to different results. Integrating this qualitative change in input factors presupposes the existence of datasets in the inventory databases.

Frankowska et al. [102] took a closer look at the different supply chain stages. They investigated different fruits and concluded that depending on the fruits and the specific handling processes, such as production in greenhouses, transport distance, or need for cooling in shops, the impact varies among the different impact categories. Bosona and Gebresenbet [123] also highlighted the importance of including various stages of the supply chain of an agricultural product in the environmental impact assessment, pointing out the great importance of waste and food losses. The combination of this holistic view on agricultural products with changing framework conditions in the future can provide guidance to policymakers and industry. Others have taken an in-depth look at the effects of ultrasonic humidification on the environmental impact of selected fruits and vegetables [124], also identifying hotspots of environmental burden along the agri-food supply chain and finding great differences between products. Therefore, one can conclude that changes in environmental impacts can be triggered by production methods, as well as handling along the supply chain, making assessment of the individual stages of the supply chain very insightful.

LCA, especially in the agri-food sector, is highly dependent on region-specific characteristics when focusing on the production stage, which subsequently influences the product quality and therewith the effectiveness of further product treatment. Nevertheless, this very individual product-specific assessment of LCA, also in the sense of production site, can be combined with future framework conditions forming the broader context.

The contribution of this study is the integration of future aspects into LCA, as well as social, economic, and political framework conditions, in addition to the technological and environmental perspective of LCA. Furthermore, “the approach meets demands to link macro scenarios into the micro or product level of LCA to help to increase the robustness of the assessments” [34]. The use of prospective LCAs can lead “to new insights during the development of new technologies, and can support policymakers in their work” [33]. Furthermore, Zimmermann et al. confirmed the increased robustness of analysis compared to conventional LCA [125]. Although external variables cannot be influenced “by technology developers, yet such variables can affect future developments to a great extent,” e.g., socioeconomic trends can shape the acceptance of new technologies [47].

## Conclusion

In this study, we developed an approach to combine LCA and qualitative scenarios to put LCA into a future context. By this means, the effects of changing framework conditions on environmental impact assessment can be made visible. This approach was applied to the LCA of a technology developed in the project FOX for the production of apple juice. Qualitative scenarios were elaborated for the European agri-food sector for the year 2035 in the same research project.

By combining these two approaches, on the one hand, it became clear that the adoption of this new small-scale technology, producing FOX apple juice (nonthermal treatment with pulsed electric field technology) is more beneficial than producing conventional apple juice, concerning the LCA results of the three different futures. In other words, environmental improvements depend on the framework conditions in which the technology is embedded. However, it has to be mentioned that this outcome is highly dependent on the scenarios used for the analysis. As the applied framework scenarios supposed a higher degree of sustainability in all of the studied futures, the results have to be interpreted in this sense. Comparing the different scenarios in which FOX apple juice can be produced, it became apparent that in the scenario “society,” society considerately enjoys a sustainable and healthy lifestyle, with society living “Greta’s dream” appearing to be the most environmental friendly, followed by the scenario “policy,” in which welfare states centrally ensure national food security and policy secures sustainability, and, lastly, by the scenario “retail,” in which markets and technologies ensure prosperity for top performers’ currency, and retailers draw the “big picture.”

On the other hand, an even greater impact can be allocated to the altered framework conditions themselves; as for both FOX and conventional apple juice, the environmental impact was reduced in all three scenarios compared to the present situation. This highlights the importance of economic and societal changes alongside technological progress.

The food system has the simultaneous inherent challenge and opportunity of being indispensable. Is the wording of the food system as a “major driver of climate change, changes in land use, depletion of freshwater resources, and pollution of aquatic and terrestrial ecosystems” [38] goal-oriented, or should we rather focus on specific aspects within the food system, which entail avoidable burdens for the environment, society, and local economy? As confirmed by Zdravkovic et al. [42], the cultivation of apples and the energy use for juice processing convey the largest part of environmental impact, independent of the production line when comparing two local solutions. However, FOX technology worked “20% more environmentally friendly than the similar small-scale stationary apple juice processing line,” while changes in transportation and operation only contributed up to 5% [42]. This highlights the importance of identifying the most important levers for environmental impact while, at the same time, focusing on those that have the greatest potential to be improved with an adequate input-output ratio, taking into consideration changes in future opportunities or risks.

## Data Availability

The datasets used and/or analyzed during the current study are available from the corresponding author upon reasonable request.

## Abbreviations

BMU: Federal Ministry for the Environment, Nature Conservation and Nuclear Safety
ESS: Ecosystem services
FOX: FOod processing in a boX
LCA: Life cycle assessment
LCC: Life cycle costing
LCI: Life cycle inventory
LCIA: Life cycle impact assessment
LCSA: Life cycle sustainability assessment
SETAC: Society of Environmental Toxicology and Chemistry
SDGs: Sustainable Development Goals
S-LCA: Social life cycle assessment
SOM: Soil organic matter

## Footnotes

1 FOod processing in a boX, European Union’s Horizon 2020 research and innovation program

2 Details on the technical equipment can be found in the work of Zdravkovic et al. [42]. For further information on the project “Innovative downscaled FOod processing in a boX” (FOX), please visit the official website: https://www.fox-foodprocessinginabox.eu/.

3 Society of Environmental Toxicology and Chemistry

4 μPt are the points expressing the total environmental impact presented as a single score in which characterization, normalization, and weighting are combined and taken into account.
